# Supplementary material for: Dose finding studies for therapies with late‐onset toxicities: A comparison study of designs
Source: Stat Med. 2022 Oct 17;41(30):5767–88. doi: 10.1002/sim.9593 (PMC10092569; doi:10.1002/sim.9593)
Supplement: Supplementary file 1 — Appendix S1 Supplementary Material [file SIM-41-5767-s001.pdf]

Supplementary material for 'Dose Finding Studies for Therapies with Late-Onset Toxicities: A Comparison Study of Designs'

1 Scenarios

Scenario	1.5MBq	2.5MBq	3.5MBq	4.5MBq	6MBq	7MBq
1 (A)	**0.30**	0.40	0.50	0.60	0.70	0.80
2	0.20	**0.30**	0.40	0.50	0.60	0.70
3	0.10	0.20	**0.30**	0.40	0.50	0.60
4	0.05	0.10	0.20	**0.30**	0.40	0.50
5	0.05	0.10	0.15	0.20	**0.30**	0.40
6	0.02	0.05	0.10	0.15	0.20	**0.30**
7 (B)	0.15	0.20	0.25	**0.30**	0.45	0.60
8	0.05	0.15	**0.30**	0.35	0.40	0.45
9 (C)	0.40	0.45	0.50	0.55	0.60	0.65
10	0.05	0.15	**0.25**	0.35	0.45	0.55
11	0.15	**0.20**	0.35	0.40	0.45	0.50
12	0.05	0.10	0.15	0.20	**0.25**	0.40
13	0.06	0.07	0.08	0.09	0.11	0.12
14	0.10	0.14	0.21	**0.30**	0.46	0.58
15	0.16	**0.30**	0.50	0.70	0.89	0.95
16	0.55	0.91	0.99	1.00	1.00	1.00
17 (D)	0.05	0.05	**0.05**	0.80	0.80	0.80

Table 1: P(DLT) in cycle 1 for each dose across 17 defined scenarios. MTD in **bold**.

Scenario	1.5MBq	2.5MBq	3.5MBq	4.5MBq	6MBq	7MBq
1 (A)	0.289	0.385	0.433	0.457	0.481	0.506
2	0.193	0.289	0.385	0.433	0.457	0.481
3	0.096	0.193	0.289	0.385	0.433	0.457
4	0.048	0.096	0.193	0.289	0.385	0.433
5	0.048	0.096	0.145	0.193	0.289	0.385
6	0.019	0.048	0.096	0.145	0.193	0.289
7 (B)	0.145	0.193	0.241	0.289	0.409	0.457
8	0.048	0.145	0.289	0.337	0.385	0.409
9 (C)	0.385	0.409	0.433	0.445	0.457	0.469
10	0.048	0.145	0.241	0.337	0.409	0.445
11	0.145	0.193	0.337	0.385	0.409	0.433
12	0.048	0.096	0.145	0.193	0.241	0.385
13	0.058	0.067	0.077	0.087	0.106	0.116
14	0.096	0.135	0.202	0.289	0.414	0.452
15	0.154	0.289	0.433	0.481	0.528	0.542
16	0.445	0.532	0.552	0.554	0.554	0.554
17 (D)	0.048	0.048	0.048	0.506	0.506	0.506

Table 2: E(nTTP) in cycle 1 for each dose across 17 defined scenarios

Scenario	1.5MBq	2.5MBq	3.5MBq	4.5MBq	6MBq	7MBq
1 (A)	0.391	0.503	0.606	0.701	0.788	0.866
2	0.270	0.391	0.503	0.606	0.701	0.788
3	0.140	0.270	0.391	0.503	0.606	0.701
4	0.071	0.140	0.270	0.391	0.503	0.606
5	0.071	0.140	0.206	0.270	0.391	0.503
6	0.029	0.071	0.140	0.206	0.270	0.391
7 (B)	0.206	0.270	0.332	0.391	0.556	0.701
8	0.071	0.206	0.391	0.448	0.503	0.556
9 (C)	0.503	0.556	0.606	0.655	0.701	0.746
10	0.071	0.206	0.332	0.448	0.556	0.655
11	0.206	0.270	0.448	0.503	0.556	0.606
12	0.071	0.140	0.206	0.270	0.332	0.503
13	0.085	0.099	0.112	0.126	0.153	0.166
14	0.140	0.193	0.282	0.391	0.566	0.683
15	0.219	0.391	0.606	0.788	0.930	0.969
16	0.655	0.944	0.994	1.000	1.000	1.000
17 (D)	0.071	0.071	0.071	0.866	0.866	0.866

Table 3: P(DLT) in cycles 1-3 for each dose across 17 defined scenarios

2 Grid of Hyper-Parameter Values Used in Prior Calibration

	**Grid**
**TITE-CRM**	$\sigma^2 \in \{0.1, 0.5, 1, 1.5\}$<br>$d \in \{(0.05, 0.10, 0.15, 0.20, 0.25, 0.30), (0.05, 0.10, 0.20, 0.30, 0.50, 0.60),$<br>$(0.15, 0.20, .025, 0.30, 0.35, 0.40), (0.15, 0.20, 0.30, 0.40, 0.50, 0.70)\}$
**TITE-CRM2**	$\mu_{a_0} \in \{-1, 1\}$<br>$\sigma_{a_0}^{-2} \in \{0.1, 0.3\}$<br>$\mu_{a_1} \in \{\log(0.2), \log(0.4)\}$<br>$\sigma_{a_1}^{-2} \in \{0.1, 0.3\}$
**ICSDP**	$\pi{*}_1 \in \{0.05, 0.10, 0.15, 0.20\}$<br>$\pi{*}_J \in \{0.30, 0.40, 0.50, 0.60\}$<br>$n_0 \in \{2, 3, 4, 5, 6\}$
**POMM**	$p_1{*} \in (0.05, 0.10, 0.15, 0.20, 0.25, 0.30), (0.05, 0.1, 0.20, 0.30, 0.50, 0.70),$<br>$(0.15, 0.20, 0.25, 0.30, 0.35, 0.40), (0.15, 0.20, 0.30, 0.40, 0.50, 0.70)$<br>$n_0 \in \{2, 3, 4, 5, 6\}$<br>$p_1^{G2}/p_1 \in \{(0.00, 0.20, 0.40, 0.60, 0.80, 1.00), (0.05, 0.10, 0.15, 0.20, 0.25, 0.30),$<br>$(0.10, 0.20, 0.30, 0.50, 0.70, 0.90), (0.20, 0.30, 0.40, 0.50, 0.60, 0.70)\}$
**nTTP**	$\mu_{\beta_0} \in \{0.0, 0.1\}$<br>$\sigma_{\beta_0}^2 \in \{10, 100, 100\}$<br>$\mu_{\beta_1} \in \{0.05, 0.50\}$<br>$\sigma_{\beta_1}^2 \in \{10, 100, 100\}$<br>$\mu_{\beta_2} \in \{0\}$<br>$\sigma_{\beta_2}^2 \in \{10, 100, 100\}$
**TITE-BOIN**	$\tau_1 \in \{0.2346, 0.2737, 0.3128, 0.3519\}$<br>$\tau_2 \in \{0.4301, 0.4692, 0.5083, 0.5474\}$<br>$\frac{\alpha}{\alpha+\beta} \in \{0.1, 0.1955, 0.391, 0.5\}$<br>$\alpha + \beta \in \{1, 2\}$
**TITE-mTPI2**	$\tau_1 \in \{0.2346, 0.2737, 0.3128, 0.3519\}$<br>$\tau_2 \in \{0.4301, 0.4692, 0.5083, 0.5474\}$
**R-mTPI2**	$\tau_1 \in \{0.2346, 0.2737, 0.3128, 0.3519\}$<br>$\tau_2 \in \{0.4301, 0.4692, 0.5083, 0.5474\}$

Table 4: The grids of hyper-parameters searched over for the prior callibration procedure.

3 Additional Results Tables

	TITECRM	TITECRM2	ICSDP	POMM	nTTP	TITEBOIN	TITE_mTPI2	R_mTPI2	Benchmark P(DLT)	Benchmark E(nTTP)
1 (A)	80	87	55	41	82	52	67	61	71	98
2	31	26	43	19	40	28	34	39	51	68
3	32	29	39	36	32	24	33	36	51	68
4	35	38	62	67	45	22	30	28	51	68
5	23	22	25	34	32	8	20	13	51	68
6	85	76	57	42	77	61	45	25	78	70
7 (B)	27	25	47	62	23	23	24	15	50	46
8	28	29	31	24	35	20	32	38	43	78
9 (C)										
10	28	30	30	18	27	20	33	32	42	72
11	25	24	26	5	27	20	32	27	32	51
12	21	21	26	29	27	6	20	11	50	78
13										
14	36	37	59	72	40	28	32	25	61	66
15	46	40	64	32	53	48	52	55	77	85
16										
17 (D)	66	77	62	34	45	88	93	83	97	51
Mean	40	40	45	37	42	32	39	35	58	69

Table 5: Setting 1: Percentage of simulations correctly identifying MTD. Scenarios 9, 13 and 16 results are not included as there is no stopping rule in Setting 1 implemented to allow for a correct result.

	TITECRM	TITECRM2	ICSDP	POMM	nTTP	TITEBOIN	TITE_mTPI2	R_mTPI2
Scenario	*Mean Duration in Weeks (sd)*							
1 (A)	39 (11)	36 (10)	46 (10)	52 (9)	32 (5)	40 (14)	46 (23)	50 (26)
2	47 (14)	44 (13)	51 (9)	54 (8)	36 (8)	48 (14)	60 (26)	69 (28)
3	57 (11)	56 (13)	54 (9)	53 (7)	45 (12)	55 (12)	78 (25)	91 (27)
4	61 (9)	62 (9)	55 (9)	54 (7)	53 (12)	59 (11)	96 (24)	109 (25)
5	60 (9)	62 (9)	58 (9)	58 (8)	56 (12)	60 (11)	106 (24)	121 (25)
6	58 (7)	61 (7)	58 (8)	61 (8)	55 (9)	56 (10)	116 (18)	132 (20)
7 (B)	55 (14)	53 (15)	55 (10)	55 (8)	44 (14)	57 (13)	81 (31)	95 (34)
8	59 (9)	59 (10)	55 (9)	54 (7)	49 (11)	56 (12)	85 (25)	96 (27)
9 (C)	34 (9)	32 (7)	44 (10)	52 (9)	31 (4)	35 (16)	37 (22)	37 (23)
10	61 (10)	60 (10)	54 (9)	53 (7)	50 (12)	57 (12)	87 (24)	100 (26)
11	54 (13)	52 (15)	54 (9)	54 (7)	42 (12)	53 (13)	73 (27)	85 (29)
12	60 (9)	62 (9)	58 (9)	59 (9)	56 (12)	60 (11)	108 (24)	123 (25)
13	57 (8)	59 (7)	58 (7)	62 (7)	52 (10)	50 (7)	117 (16)	141 (17)
14	60 (11)	59 (12)	55 (9)	54 (7)	50 (14)	59 (11)	90 (26)	106 (28)
15	48 (12)	45 (11)	51 (8)	54 (7)	37 (8)	45 (10)	59 (19)	70 (22)
16	29 (4)	29 (4)	36 (5)	44 (5)	29 (4)	22 (6)	24 (9)	23 (11)
17 (D)	63 (7)	57 (8)	57 (5)	55 (6)	56 (10)	49 (3)	85 (7)	100 (9)
Mean	53	52	53	55	46	51	79	91
Scenario	*Mean Number of Patients (sd)*							
1 (A)	14 (6)	12 (5)	17 (5)	20 (4)	10 (2)	19 (6)	15 (5)	15 (5)
2	18 (7)	16 (7)	20 (5)	21 (4)	12 (4)	23 (5)	18 (5)	18 (6)
3	23 (6)	22 (6)	21 (4)	21 (3)	17 (6)	26 (4)	21 (5)	22 (6)
4	25 (5)	25 (5)	22 (5)	21 (3)	21 (6)	27 (3)	24 (4)	25 (5)
5	24 (4)	25 (5)	23 (5)	23 (4)	22 (6)	27 (3)	25 (4)	27 (4)
6	23 (4)	25 (3)	23 (4)	25 (4)	22 (5)	26 (3)	26 (3)	28 (3)
7 (B)	22 (7)	21 (8)	22 (5)	22 (4)	16 (7)	26 (4)	21 (6)	23 (7)
8	24 (5)	24 (5)	21 (4)	21 (3)	19 (6)	26 (4)	22 (4)	23 (5)
9 (C)	11 (4)	10 (3)	16 (5)	20 (4)	10 (2)	17 (7)	13 (5)	12 (5)
10	24 (5)	24 (5)	21 (4)	21 (3)	19 (6)	27 (3)	23 (4)	24 (5)
11	21 (7)	20 (7)	21 (5)	21 (4)	15 (6)	25 (4)	20 (5)	21 (6)
12	24 (4)	25 (4)	23 (5)	24 (4)	22 (6)	27 (3)	25 (4)	27 (4)
13	22 (4)	23 (4)	23 (3)	25 (3)	20 (5)	25 (2)	24 (3)	27 (3)
14	24 (6)	24 (6)	22 (5)	21 (4)	19 (7)	27 (3)	23 (5)	25 (5)
15	18 (6)	17 (6)	20 (4)	21 (4)	13 (4)	22 (4)	18 (4)	18 (5)
16	9 (1)	9 (1)	14 (3)	17 (2)	9 (1)	11 (3)	10 (2)	10 (2)
17 (D)	26 (4)	23 (4)	23 (3)	22 (2)	23 (5)	24 (1)	24 (2)	25 (2)
Mean	21	20	21	22	17	24	21	22

Table 6: Setting 1: Measures of size of the trial across scenarios, mean and sd of duration and number of patients.

	**TITECRM**	**TITECRM2**	**ICSDP**	**POMM**	**nTTP**	**TITEBOIN**	**TITE_mTPI2**	**R_mTPI2**
Scenario	*Mean Number of Patients on MTD (sd)*							
1 (A)	8 (2)	9 (1)	7 (3)	7 (3)	8 (2)	7 (3)	7 (3)	8 (2)
2	5 (4)	4 (4)	6 (3)	5 (3)	4 (4)	6 (3)	6 (3)	6 (3)
3	5 (4)	5 (4)	5 (4)	5 (3)	4 (4)	6 (3)	6 (4)	6 (3)
4	6 (3)	6 (3)	8 (2)	8 (3)	6 (4)	6 (3)	6 (4)	6 (3)
5	4 (4)	4 (4)	5 (4)	5 (4)	4 (4)	6 (3)	5 (4)	5 (4)
6	8 (2)	7 (3)	6 (4)	6 (4)	7 (3)	8 (3)	6 (4)	5 (4)
7 (B)	4 (4)	4 (4)	6 (3)	7 (3)	3 (4)	6 (3)	4 (4)	4 (4)
8	5 (3)	5 (4)	4 (4)	4 (3)	4 (4)	6 (3)	6 (3)	7 (3)
9 (C)	NA (NA)	NA (NA)	NA (NA)	NA (NA)	NA (NA)	NA (NA)	NA (NA)	NA (NA)
10	5 (3)	5 (3)	4 (4)	4 (3)	4 (4)	6 (3)	6 (3)	7 (3)
11	5 (3)	5 (3)	5 (3)	4 (2)	4 (4)	5 (3)	6 (3)	6 (3)
12	4 (4)	4 (3)	5 (4)	5 (4)	4 (4)	6 (3)	5 (4)	5 (3)
13	NA (NA)	NA (NA)	NA (NA)	NA (NA)	NA (NA)	NA (NA)	NA (NA)	NA (NA)
14	5 (4)	5 (4)	7 (3)	8 (3)	5 (4)	6 (3)	5 (4)	6 (4)
15	6 (3)	6 (4)	8 (2)	6 (3)	5 (4)	8 (2)	7 (3)	7 (3)
16	NA (NA)	NA (NA)	NA (NA)	NA (NA)	NA (NA)	NA (NA)	NA (NA)	NA (NA)
17 (D)	8 (2)	8 (3)	8 (2)	7 (2)	7 (3)	9 (0)	9 (1)	9 (1)
Mean	6	5	6	6	5	6	6	6
Scenario	*Mean Number of Patients on Unsafe Doses (sd)*							
1 (A)	5 (7)	4 (5)	10 (6)	14 (5)	2 (4)	13 (8)	7 (7)	7 (7)
2	6 (7)	4 (6)	8 (6)	12 (5)	1 (4)	12 (8)	6 (7)	6 (6)
3	8 (6)	6 (6)	8 (5)	9 (4)	3 (5)	11 (7)	6 (6)	5 (6)
4	8 (5)	6 (5)	5 (5)	4 (5)	5 (5)	9 (6)	5 (6)	4 (5)
5	6 (4)	5 (4)	4 (4)	3 (4)	4 (4)	6 (4)	4 (4)	3 (4)
6	0 (0)	0 (0)	0 (0)	0 (0)	0 (0)	0 (0)	0 (0)	0 (0)
7 (B)	4 (5)	3 (4)	3 (4)	3 (4)	2 (4)	8 (6)	4 (5)	3 (4)
8	10 (6)	9 (6)	10 (6)	10 (5)	6 (6)	12 (7)	7 (7)	6 (6)
9 (C)	11 (4)	10 (3)	16 (5)	20 (4)	10 (2)	17 (7)	13 (5)	12 (5)
10	11 (6)	9 (6)	10 (5)	10 (4)	7 (6)	13 (7)	8 (7)	8 (6)
11	10 (7)	8 (7)	12 (6)	13 (4)	5 (6)	16 (7)	10 (7)	10 (7)
12	6 (4)	5 (4)	4 (4)	4 (4)	5 (4)	7 (3)	4 (4)	3 (4)
13	0 (0)	0 (0)	0 (0)	0 (0)	0 (0)	0 (0)	0 (0)	0 (0)
14	6 (5)	4 (5)	4 (4)	3 (4)	3 (4)	8 (6)	4 (5)	4 (4)
15	5 (5)	4 (4)	7 (5)	10 (4)	1 (3)	9 (6)	5 (5)	5 (5)
16	9 (1)	9 (1)	14 (3)	17 (2)	9 (1)	11 (3)	10 (2)	10 (2)
17 (D)	10 (3)	7 (3)	8 (2)	9 (1)	8 (4)	9 (1)	9 (2)	8 (1)
Mean	7	6	7	8	4	10	6	5

Table 7: Setting 1: Mean and standard deviation of the number of patients assigned to the true MTD and to unsafe doses.

	TITECRM	TITECRM2	ICSDP	POMM	nTTP	TITEBOIN	TITE_mTPI2	R_mTPI2
Stopping Reason				*Scenario 1 (A)*				
Sufficient Information	99	100	100	97	100	95	100	99
Lowest Dose Deemed Unsafe	0	0	0	0	0	0	0	0
Highest Dose Deemed too Safe	0	0	0	0	0	0	0	0
Precision	0	0	0	0	0	0	0	0
Hard Safety	0	0	0	0	0	0	0	0
Maximum Patients	2	1	2	3	0	10	1	1
Unsafe (Total)	0	0	0	0	0	0	0	0
Sufficient/Precision (Total)	99	100	100	97	100	95	100	99
Stopping Reason				*Scenario 2*				
Sufficient Information	97	97	99	96	100	89	98	96
Lowest Dose Deemed Unsafe	0	0	0	0	0	0	0	0
Highest Dose Deemed too Safe	0	0	0	0	0	0	0	0
Precision	0	0	0	0	0	0	0	0
Hard Safety	0	0	0	0	0	0	0	0
Maximum Patients	7	7	4	4	0	19	4	6
Unsafe (Total)	0	0	0	0	0	0	0	0
Sufficient/Precision (Total)	97	97	99	96	100	89	98	96
Stopping Reason				*Scenario 3*				
Sufficient Information	91	91	98	97	98	83	97	86
Lowest Dose Deemed Unsafe	0	0	0	0	0	0	0	0
Highest Dose Deemed too Safe	0	0	0	0	0	0	0	0
Precision	0	0	0	0	0	0	0	0
Hard Safety	0	0	0	0	0	0	0	0
Maximum Patients	20	20	6	3	1	32	7	19
Unsafe (Total)	0	0	0	0	0	0	0	0
Sufficient/Precision (Total)	91	91	98	97	98	83	97	86
Stopping Reason				*Scenario 4*				
Sufficient Information	86	86	97	97	95	82	94	73
Lowest Dose Deemed Unsafe	0	0	0	0	0	0	0	0
Highest Dose Deemed too Safe	0	0	0	0	0	0	0	0
Precision	0	0	0	0	0	0	0	0
Hard Safety	0	0	0	0	0	0	0	0
Maximum Patients	30	29	9	3	5	43	14	36
Unsafe (Total)	0	0	0	0	0	0	0	0
Sufficient/Precision (Total)	86	86	97	97	95	82	94	73
Stopping Reason				*Scenario 5*				
Sufficient Information	89	84	95	86	91	85	93	57
Lowest Dose Deemed Unsafe	0	0	0	0	0	0	0	0
Highest Dose Deemed too Safe	0	0	0	0	0	0	0	0
Precision	0	0	0	0	0	0	0	0
Hard Safety	0	0	0	0	0	0	0	0
Maximum Patients	24	32	15	14	9	44	21	53
Unsafe (Total)	0	0	0	0	0	0	0	0
Sufficient/Precision (Total)	89	84	95	86	91	85	93	57
Stopping Reason				*Scenario 6*				
Sufficient Information	95	91	97	79	95	92	93	55
Lowest Dose Deemed Unsafe	0	0	0	0	0	0	0	0
Highest Dose Deemed too Safe	0	0	0	0	0	0	0	0
Precision	0	0	0	0	0	0	0	0
Hard Safety	0	0	0	0	0	0	0	0
Maximum Patients	11	20	12	21	5	30	22	52
Unsafe (Total)	0	0	0	0	0	0	0	0
Sufficient/Precision (Total)	95	91	97	79	95	92	93	55

Table 8: Setting 1: Summary of stopping reasons for Setting 1 (Scenarios 1-6), expressed as a percentage of simulations where the given stopping rule was triggered. The sum of these may be greater than 100, since it is possible for more than one rule to be triggered in a single trial.

	TITECRM	TITECRM2	ICSDP	POMM	nTTP	TITEBOIN	TITE_mTPI2	R_mTPI2
Stopping Reason					*Scenario 7 (B)*			
Sufficient Information	90	88	95	95	96	80	95	75
Lowest Dose Deemed Unsafe	0	0	0	0	0	0	0	0
Highest Dose Deemed too Safe	0	0	0	0	0	0	0	0
Precision	0	0	0	0	0	0	0	0
Hard Safety	0	0	0	0	0	0	0	0
Maximum Patients	21	22	11	5	4	41	11	32
Unsafe (Total)	0	0	0	0	0	0	0	0
Sufficient/Precision (Total)	90	88	95	95	96	80	95	75
Stopping Reason					*Scenario 8*			
Sufficient Information	91	89	97	97	97	85	96	83
Lowest Dose Deemed Unsafe	0	0	0	0	0	0	0	0
Highest Dose Deemed too Safe	0	0	0	0	0	0	0	0
Precision	0	0	0	0	0	0	0	0
Hard Safety	0	0	0	0	0	0	0	0
Maximum Patients	21	23	8	3	3	33	9	22
Unsafe (Total)	0	0	0	0	0	0	0	0
Sufficient/Precision (Total)	91	89	97	97	97	85	96	83
Stopping Reason					*Scenario 9 (C)*			
Sufficient Information	100	100	100	96	100	95	100	100
Lowest Dose Deemed Unsafe	0	0	0	0	0	0	0	0
Highest Dose Deemed too Safe	0	0	0	0	0	0	0	0
Precision	0	0	0	0	0	0	0	0
Hard Safety	0	0	0	0	0	0	0	0
Maximum Patients	1	0	1	4	0	8	1	1
Unsafe (Total)	0	0	0	0	0	0	0	0
Sufficient/Precision (Total)	100	100	100	96	100	95	100	100
Stopping Reason					*Scenario 10*			
Sufficient Information	87	89	98	98	97	82	95	81
Lowest Dose Deemed Unsafe	0	0	0	0	0	0	0	0
Highest Dose Deemed too Safe	0	0	0	0	0	0	0	0
Precision	0	0	0	0	0	0	0	0
Hard Safety	0	0	0	0	0	0	0	0
Maximum Patients	26	25	7	2	3	37	10	25
Unsafe (Total)	0	0	0	0	0	0	0	0
Sufficient/Precision (Total)	87	89	98	98	97	82	95	81
Stopping Reason					*Scenario 11*			
Sufficient Information	93	92	97	96	99	85	97	88
Lowest Dose Deemed Unsafe	0	0	0	0	0	0	0	0
Highest Dose Deemed too Safe	0	0	0	0	0	0	0	0
Precision	0	0	0	0	0	0	0	0
Hard Safety	0	0	0	0	0	0	0	0
Maximum Patients	14	17	7	4	1	29	7	16
Unsafe (Total)	0	0	0	0	0	0	0	0
Sufficient/Precision (Total)	93	92	97	96	99	85	97	88
Stopping Reason					*Scenario 12*			
Sufficient Information	89	83	94	83	90	86	92	54
Lowest Dose Deemed Unsafe	0	0	0	0	0	0	0	0
Highest Dose Deemed too Safe	0	0	0	0	0	0	0	0
Precision	0	0	0	0	0	0	0	0
Hard Safety	0	0	0	0	0	0	0	0
Maximum Patients	23	31	16	17	10	46	24	55
Unsafe (Total)	0	0	0	0	0	0	0	0
Sufficient/Precision (Total)	89	83	94	83	90	86	92	54

Table 9: Setting 1: Summary of stopping reasons for Setting 1 (Scenarios 7-12), expressed as a percentage of simulations where the given stopping rule was triggered. The sum of these may be greater than 100, since it is possible for more than one rule to be triggered in a single trial.

	TITECRM	TITECRM2	ICSDP	POMM	nTTP	TITEBOIN	TITE_mTPI2	R_mTPI2
Stopping Reason					*Scenario 13*			
Sufficient Information	95	96	97	80	98	98	94	63
Lowest Dose Deemed Unsafe	0	0	0	0	0	0	0	0
Highest Dose Deemed too Safe	0	0	0	0	0	0	0	0
Precision	0	0	0	0	0	0	0	0
Hard Safety	0	0	0	0	0	0	0	0
Maximum Patients	8	8	8	20	2	8	10	46
Unsafe (Total)	0	0	0	0	0	0	0	0
Sufficient/Precision (Total)	95	96	97	80	98	98	94	63
Stopping Reason					*Scenario 14*			
Sufficient Information	87	85	96	97	95	80	94	73
Lowest Dose Deemed Unsafe	0	0	0	0	0	0	0	0
Highest Dose Deemed too Safe	0	0	0	0	0	0	0	0
Precision	0	0	0	0	0	0	0	0
Hard Safety	0	0	0	0	0	0	0	0
Maximum Patients	28	28	9	3	5	42	13	37
Unsafe (Total)	0	0	0	0	0	0	0	0
Sufficient/Precision (Total)	87	85	96	97	95	80	94	73
Stopping Reason					*Scenario 15*			
Sufficient Information	99	99	100	98	100	97	100	99
Lowest Dose Deemed Unsafe	0	0	0	0	0	0	0	0
Highest Dose Deemed too Safe	0	0	0	0	0	0	0	0
Precision	0	0	0	0	0	0	0	0
Hard Safety	0	0	0	0	0	0	0	0
Maximum Patients	3	3	2	2	0	9	2	2
Unsafe (Total)	0	0	0	0	0	0	0	0
Sufficient/Precision (Total)	99	99	100	98	100	97	100	99
Stopping Reason					*Scenario 16*			
Sufficient Information	100	100	100	100	100	100	100	100
Lowest Dose Deemed Unsafe	0	0	0	0	0	0	0	0
Highest Dose Deemed too Safe	0	0	0	0	0	0	0	0
Precision	0	0	0	0	0	0	0	0
Hard Safety	0	0	0	0	0	0	0	0
Maximum Patients	0	0	0	0	0	0	0	0
Unsafe (Total)	0	0	0	0	0	0	0	0
Sufficient/Precision (Total)	100	100	100	100	100	100	100	100
Stopping Reason					*Scenario 17 (D)*			
Sufficient Information	89	94	100	99	96	99	99	99
Lowest Dose Deemed Unsafe	0	0	0	0	0	0	0	0
Highest Dose Deemed too Safe	0	0	0	0	0	0	0	0
Precision	0	0	0	0	0	0	0	0
Hard Safety	0	0	0	0	0	0	0	0
Maximum Patients	32	16	2	1	4	2	2	5
Unsafe (Total)	0	0	0	0	0	0	0	0
Sufficient/Precision (Total)	89	94	100	99	96	99	99	99

Table 10: Setting 1: Summary of stopping reasons for Setting 1 (Scenarios 13-17), expressed as a percentage of simulations where the given stopping rule was triggered. The sum of these may be greater than 100, since it is possible for more than one rule to be triggered in a single trial.

	TITECRM	TITECRM2	ICSDP	POMM	nTTP	TITEBOIN	TITE_mTPI2	R_mTPI2	Benchmark P(DLT)	Benchmark E(nTTP)
1 (A)	38	38	49	34	57	29	39	35	71	98
2	30	25	41	22	37	27	34	38	51	68
3	28	29	39	26	29	25	32	35	51	68
4	24	37	56	54	30	22	30	28	51	68
5	20	21	23	48	22	15	22	13	51	68
6	79	27	51	11	18	29	19	12	78	70
7 (B)	23	24	44	54	13	23	24	16	50	46
8	23	28	32	17	36	20	32	36	43	78
9 (C)	63	73	29	50	72	48	51	53		
10	29	31	34	17	34	21	33	34	42	72
11	26	25	28	15	30	21	33	30	32	51
12	24	21	26	50	21	16	23	12	50	78
13	36	77	28	37	59	79	72	71		
14	35	35	55	58	23	27	32	26	61	66
15	44	40	59	40	46	49	51	54	77	85
16	85	96	66	89	96	80	80	81		
17 (D)	83	82	97	62	64	97	97	97	97	51
Mean	41	42	45	40	40	37	41	39		

Table 11: Setting 2: Percentage of simulations correctly identifying MTD.

	TITECRM	TITECRM2	ICSDP	POMM	nTTP	TITEBOIN	TITE_mTPI2	R_mTPI2
Scenario	*Mean Duration in Weeks (sd)*							
1 (A)	39 (13)	31 (13)	44 (12)	44 (13)	29 (4)	33 (18)	41 (26)	43 (30)
2	48 (13)	42 (15)	50 (10)	47 (11)	30 (3)	42 (18)	57 (28)	63 (33)
3	54 (11)	53 (13)	52 (9)	44 (8)	31 (4)	50 (15)	76 (26)	88 (30)
4	51 (11)	57 (11)	52 (9)	42 (6)	32 (5)	55 (13)	93 (23)	107 (26)
5	47 (9)	57 (11)	55 (10)	43 (7)	34 (6)	54 (14)	101 (23)	117 (25)
6	44 (7)	55 (9)	56 (9)	43 (6)	35 (5)	50 (13)	108 (18)	122 (20)
7 (B)	52 (12)	50 (16)	53 (10)	45 (9)	32 (6)	51 (17)	78 (32)	90 (38)
8	53 (11)	56 (11)	52 (9)	43 (7)	32 (4)	52 (14)	83 (24)	94 (27)
9 (C)	32 (13)	25 (11)	38 (14)	39 (15)	27 (5)	27 (19)	32 (25)	29 (27)
10	60 (10)	56 (11)	52 (9)	42 (7)	32 (4)	53 (13)	85 (24)	98 (27)
11	53 (13)	49 (16)	53 (9)	46 (9)	31 (5)	48 (17)	70 (29)	80 (33)
12	58 (10)	57 (11)	56 (10)	43 (7)	34 (6)	54 (14)	102 (24)	118 (26)
13	51 (8)	51 (9)	57 (7)	47 (8)	36 (6)	41 (10)	102 (18)	115 (21)
14	59 (12)	55 (14)	52 (10)	43 (7)	33 (6)	54 (15)	87 (27)	102 (31)
15	47 (12)	43 (13)	50 (9)	48 (10)	30 (3)	40 (14)	56 (21)	65 (26)
16	22 (9)	18 (7)	25 (11)	22 (9)	23 (4)	13 (8)	16 (13)	14 (13)
17 (D)	57 (9)	50 (10)	49 (5)	36 (7)	29 (5)	40 (7)	76 (10)	92 (12)
Mean	49	48	50	42	31	44	74	84
Scenario	*Mean Number of Patients (sd)*							
1 (A)	14 (6)	10 (6)	16 (6)	17 (6)	9 (2)	16 (8)	13 (7)	12 (7)
2	18 (6)	15 (8)	19 (5)	18 (5)	9 (2)	20 (8)	17 (7)	17 (7)
3	21 (6)	21 (7)	20 (4)	16 (4)	10 (2)	24 (6)	20 (5)	21 (6)
4	20 (5)	23 (5)	20 (4)	15 (3)	10 (3)	25 (4)	23 (4)	25 (5)
5	18 (5)	23 (5)	22 (5)	16 (3)	11 (3)	25 (5)	24 (4)	26 (5)
6	16 (4)	22 (5)	22 (4)	16 (3)	11 (3)	24 (5)	24 (4)	26 (4)
7 (B)	20 (6)	19 (8)	21 (5)	17 (4)	10 (3)	24 (7)	20 (7)	22 (8)
8	21 (5)	22 (5)	20 (4)	16 (3)	10 (2)	24 (5)	21 (4)	22 (5)
9 (C)	11 (6)	8 (5)	14 (6)	14 (7)	8 (2)	13 (9)	10 (7)	9 (6)
10	24 (5)	23 (5)	20 (4)	15 (3)	10 (2)	25 (5)	22 (4)	23 (5)
11	21 (7)	19 (8)	21 (5)	17 (4)	10 (2)	23 (7)	19 (6)	20 (7)
12	23 (5)	23 (5)	22 (5)	16 (3)	11 (3)	25 (5)	24 (5)	26 (5)
13	19 (4)	19 (5)	23 (3)	17 (4)	12 (3)	20 (4)	21 (4)	24 (5)
14	24 (6)	22 (7)	21 (5)	16 (3)	11 (3)	25 (5)	22 (5)	24 (6)
15	18 (6)	16 (6)	19 (4)	18 (5)	9 (1)	19 (6)	17 (5)	17 (6)
16	7 (2)	5 (2)	9 (4)	8 (3)	7 (1)	6 (4)	6 (4)	5 (3)
17 (D)	23 (5)	20 (4)	19 (3)	13 (3)	10 (2)	20 (3)	20 (3)	21 (3)
Mean	19	18	19	16	10	21	19	20

Table 12: Setting 2: Measures of size of the trial across scenarios, mean and sd of duration and number of patients.

	**TITECRM**	**TITECRM2**	**ICSDP**	**POMM**	**nTTP**	**TITEBOIN**	**TITE_mTPI2**	**R_mTPI2**
Scenario	*Mean Number of Patients on MTD (sd)*							
1 (A)	7 (2)	7 (3)	7 (3)	6 (3)	7 (2)	5 (3)	6 (3)	6 (3)
2	6 (3)	4 (4)	6 (3)	5 (3)	3 (3)	6 (3)	5 (4)	6 (4)
3	5 (3)	4 (4)	5 (4)	4 (3)	1 (1)	6 (3)	5 (4)	6 (3)
4	5 (3)	5 (3)	7 (3)	4 (2)	2 (2)	6 (3)	5 (3)	6 (3)
5	2 (3)	3 (3)	4 (3)	2 (2)	0 (1)	6 (3)	5 (4)	5 (4)
6	2 (2)	5 (3)	6 (4)	1 (2)	2 (2)	6 (3)	4 (4)	4 (3)
7 (B)	4 (4)	3 (4)	6 (3)	4 (3)	1 (1)	5 (3)	4 (4)	4 (4)
8	5 (3)	5 (3)	4 (4)	3 (2)	1 (1)	5 (3)	6 (3)	7 (3)
9 (C)	NA (NA)	NA (NA)	NA (NA)	NA (NA)	NA (NA)	NA (NA)	NA (NA)	NA (NA)
10	5 (3)	5 (3)	4 (4)	3 (2)	1 (1)	5 (3)	6 (3)	6 (3)
11	5 (3)	5 (3)	5 (3)	4 (2)	3 (3)	5 (3)	6 (3)	6 (3)
12	4 (4)	3 (3)	4 (3)	2 (2)	0 (1)	6 (3)	5 (4)	5 (3)
13	NA (NA)	NA (NA)	NA (NA)	NA (NA)	NA (NA)	NA (NA)	NA (NA)	NA (NA)
14	5 (4)	5 (3)	7 (3)	4 (3)	1 (2)	6 (3)	5 (4)	6 (4)
15	6 (3)	5 (4)	7 (3)	6 (3)	3 (3)	7 (3)	7 (3)	7 (3)
16	NA (NA)	NA (NA)	NA (NA)	NA (NA)	NA (NA)	NA (NA)	NA (NA)	NA (NA)
17 (D)	9 (2)	7 (3)	7 (3)	4 (2)	1 (1)	9 (1)	9 (1)	9 (1)
Mean	5	5	6	4	2	6	6	6
Scenario	*Mean Number of Patients on Unsafe Doses (sd)*							
1 (A)	7 (6)	4 (5)	10 (7)	10 (6)	2 (3)	11 (8)	7 (7)	6 (6)
2	6 (6)	4 (6)	8 (6)	8 (4)	0 (1)	10 (8)	6 (7)	5 (6)
3	6 (5)	6 (5)	7 (5)	5 (3)	1 (2)	10 (7)	5 (6)	5 (6)
4	4 (4)	5 (5)	4 (5)	2 (3)	1 (1)	8 (6)	5 (5)	4 (5)
5	2 (2)	4 (3)	4 (4)	1 (2)	1 (2)	5 (3)	3 (3)	2 (3)
6	0 (0)	0 (0)	0 (0)	0 (0)	0 (0)	0 (0)	0 (0)	0 (0)
7 (B)	2 (3)	3 (4)	3 (4)	2 (3)	0 (1)	7 (6)	3 (5)	2 (4)
8	8 (5)	8 (6)	9 (5)	6 (3)	1 (2)	11 (7)	7 (7)	6 (6)
9 (C)	11 (6)	8 (5)	14 (6)	14 (7)	8 (2)	13 (9)	10 (7)	9 (6)
10	11 (6)	8 (5)	9 (5)	6 (3)	2 (2)	12 (7)	8 (7)	7 (6)
11	10 (7)	8 (7)	11 (5)	9 (4)	1 (2)	14 (7)	9 (7)	9 (7)
12	5 (4)	4 (3)	4 (4)	1 (2)	1 (2)	5 (3)	3 (3)	3 (3)
13	0 (0)	0 (0)	0 (0)	0 (0)	0 (0)	0 (0)	0 (0)	0 (0)
14	5 (5)	4 (4)	4 (4)	2 (2)	1 (1)	7 (6)	4 (5)	3 (4)
15	5 (5)	3 (4)	7 (4)	7 (4)	0 (1)	8 (6)	5 (5)	4 (5)
16	7 (2)	5 (2)	9 (4)	8 (3)	7 (1)	6 (4)	6 (4)	5 (3)
17 (D)	7 (4)	5 (2)	5 (2)	3 (1)	2 (1)	5 (3)	5 (2)	5 (2)
Mean	6	5	6	5	2	8	5	4

Table 13: Setting 2: Mean and standard deviation of the number of patients assigned to the true MTD and to unsafe doses.

	TITECRM	TITECRM2	ICSDP	POMM	nTTP	TITEBOIN	TITE_mTPI2	R_mTPI2
Stopping Reason				*Scenario 1 (A)*				
Sufficient Information	57	46	88	60	46	68	70	68
Lowest Dose Deemed Unsafe	42	50	11	20	22	28	30	32
Highest Dose Deemed too Safe	0	0	0	0	0	0	0	0
Precision	0	17	2	14	73	0	0	0
Hard Safety	10	4	11	14	10	3	4	4
Maximum Patients	2	1	1	2	0	7	1	1
Unsafe (Total)	42	50	11	26	25	28	30	32
Sufficient/Precision (Total)	58	50	89	71	75	68	70	68
Stopping Reason				*Scenario 2*				
Sufficient Information	78	68	91	57	43	80	86	82
Lowest Dose Deemed Unsafe	16	23	2	5	3	12	13	15
Highest Dose Deemed too Safe	0	0	0	0	0	0	0	0
Precision	4	15	9	42	92	0	0	0
Hard Safety	2	1	2	4	2	1	1	1
Maximum Patients	6	5	3	2	0	15	3	5
Unsafe (Total)	16	23	2	7	5	12	13	15
Sufficient/Precision (Total)	82	75	97	91	95	80	86	82
Stopping Reason				*Scenario 3*				
Sufficient Information	75	73	82	34	20	82	94	83
Lowest Dose Deemed Unsafe	2	5	0	0	0	3	3	3
Highest Dose Deemed too Safe	0	1	0	0	0	2	0	0
Precision	23	22	25	78	99	0	0	0
Hard Safety	0	0	0	0	0	0	0	0
Maximum Patients	11	15	4	1	0	25	6	17
Unsafe (Total)	2	5	0	1	1	3	3	3
Sufficient/Precision (Total)	94	87	98	99	99	82	94	83
Stopping Reason				*Scenario 4*				
Sufficient Information	44	58	71	17	9	78	91	72
Lowest Dose Deemed Unsafe	0	1	0	0	0	1	1	1
Highest Dose Deemed too Safe	0	6	0	0	1	7	4	2
Precision	61	36	39	92	99	0	0	0
Hard Safety	0	0	0	0	0	0	0	0
Maximum Patients	7	17	6	0	0	32	11	32
Unsafe (Total)	0	1	0	0	0	1	1	1
Sufficient/Precision (Total)	98	85	97	100	100	78	91	72
Stopping Reason				*Scenario 5*				
Sufficient Information	22	49	73	14	9	69	82	55
Lowest Dose Deemed Unsafe	0	1	0	0	0	1	1	1
Highest Dose Deemed too Safe	2	19	1	1	5	20	12	9
Precision	83	26	31	92	97	0	0	0
Hard Safety	0	0	0	0	0	0	0	0
Maximum Patients	2	20	11	1	0	31	15	44
Unsafe (Total)	0	1	0	0	0	1	1	1
Sufficient/Precision (Total)	100	69	95	99	97	69	82	55
Stopping Reason				*Scenario 6*				
Sufficient Information	7	37	78	8	3	57	66	41
Lowest Dose Deemed Unsafe	0	0	0	0	0	0	0	0
Highest Dose Deemed too Safe	8	45	3	4	26	37	30	29
Precision	95	17	22	93	85	0	0	0
Hard Safety	0	0	0	0	0	0	0	0
Maximum Patients	0	11	9	0	0	21	15	38
Unsafe (Total)	0	0	0	0	0	0	0	0
Sufficient/Precision (Total)	100	50	94	96	85	57	66	41

Table 14: Setting 2: Summary of stopping reasons for Setting 2 (Scenarios 1-6), expressed as a percentage of simulations where the given stopping rule was triggered. The sum of these may be greater than 100, since it is possible for more than one rule to be triggered in a single trial.

	TITECRM	TITECRM2	ICSDP	POMM	nTTP	TITEBOIN	TITE_mTPI2	R_mTPI2
Stopping Reason				*Scenario 7 (B)*				
Sufficient Information	65	65	83	35	32	77	89	70
Lowest Dose Deemed Unsafe	4	11	1	1	1	6	6	8
Highest Dose Deemed too Safe	0	1	0	0	0	2	1	1
Precision	34	21	21	72	96	0	0	0
Hard Safety	1	0	1	1	1	0	0	0
Maximum Patients	7	17	9	2	0	29	8	27
Unsafe (Total)	4	11	1	2	1	6	6	8
Sufficient/Precision (Total)	94	79	95	97	98	77	89	70
Stopping Reason				*Scenario 8*				
Sufficient Information	59	70	78	26	9	79	93	82
Lowest Dose Deemed Unsafe	0	1	0	0	0	1	1	1
Highest Dose Deemed too Safe	0	6	0	0	1	8	3	1
Precision	42	24	31	86	100	0	0	0
Hard Safety	0	0	0	0	0	0	0	0
Maximum Patients	8	16	6	0	0	26	8	20
Unsafe (Total)	0	1	0	0	0	1	1	1
Sufficient/Precision (Total)	97	86	98	99	100	79	93	82
Stopping Reason				*Scenario 9 (C)*				
Sufficient Information	37	24	70	42	20	49	49	47
Lowest Dose Deemed Unsafe	63	73	29	38	71	48	51	53
Highest Dose Deemed too Safe	0	0	0	0	0	0	0	0
Precision	0	19	1	6	32	0	0	0
Hard Safety	24	10	29	30	21	9	10	10
Maximum Patients	1	0	1	2	0	6	1	0
Unsafe (Total)	63	73	29	50	72	48	51	53
Sufficient/Precision (Total)	37	27	71	48	28	49	49	47
Stopping Reason				*Scenario 10*				
Sufficient Information	85	68	76	23	9	81	93	81
Lowest Dose Deemed Unsafe	1	1	0	0	0	1	1	1
Highest Dose Deemed too Safe	1	3	0	0	0	4	2	1
Precision	4	30	35	88	99	0	0	0
Hard Safety	0	0	0	0	0	0	0	0
Maximum Patients	25	16	4	0	0	29	9	23
Unsafe (Total)	1	1	0	0	0	1	1	1
Sufficient/Precision (Total)	88	89	98	100	100	81	93	81
Stopping Reason				*Scenario 11*				
Sufficient Information	89	71	86	42	33	78	90	81
Lowest Dose Deemed Unsafe	4	11	1	1	1	6	6	8
Highest Dose Deemed too Safe	0	2	0	0	0	4	1	0
Precision	1	16	18	66	97	0	0	0
Hard Safety	1	0	1	1	1	0	0	0
Maximum Patients	14	13	6	2	0	22	6	13
Unsafe (Total)	4	11	1	2	1	6	6	8
Sufficient/Precision (Total)	90	81	97	96	98	78	90	81
Stopping Reason				*Scenario 12*				
Sufficient Information	71	48	75	14	9	69	80	51
Lowest Dose Deemed Unsafe	0	1	0	0	0	1	1	1
Highest Dose Deemed too Safe	8	21	1	1	6	21	14	11
Precision	28	24	28	91	97	0	0	0
Hard Safety	0	0	0	0	0	0	0	0
Maximum Patients	20	21	12	1	0	31	17	46
Unsafe (Total)	0	1	0	0	0	1	1	1
Sufficient/Precision (Total)	92	67	95	98	97	69	80	51

Table 15: Setting 2: Summary of stopping reasons for Setting 2 (Scenarios 7-12), expressed as a percentage of simulations where the given stopping rule was triggered. The sum of these may be greater than 100, since it is possible for more than one rule to be triggered in a single trial.

	TITECRM	TITECRM2	ICSDP	POMM	nTTP	TITEBOIN	TITE_mTPI2	R_mTPI2
Stopping Reason				*Scenario 13*				
Sufficient Information	34	18	68	11	10	19	25	14
Lowest Dose Deemed Unsafe	0	1	0	0	0	1	1	1
Highest Dose Deemed too Safe	36	77	28	37	59	79	72	71
Precision	74	1	2	58	45	0	0	0
Hard Safety	0	0	0	0	0	0	0	0
Maximum Patients	4	5	7	1	0	5	4	20
Unsafe (Total)	0	1	0	0	0	1	1	1
Sufficient/Precision (Total)	96	19	70	67	45	19	25	14
Stopping Reason				*Scenario 14*				
Sufficient Information	84	61	76	25	20	78	91	72
Lowest Dose Deemed Unsafe	1	4	0	0	0	3	3	3
Highest Dose Deemed too Safe	1	3	0	0	0	3	1	1
Precision	4	30	32	85	99	0	0	0
Hard Safety	0	0	0	0	0	0	0	0
Maximum Patients	25	19	7	1	0	31	10	32
Unsafe (Total)	1	4	0	0	0	3	3	3
Sufficient/Precision (Total)	88	82	97	99	99	78	91	72
Stopping Reason				*Scenario 15*				
Sufficient Information	91	77	94	64	36	90	92	90
Lowest Dose Deemed Unsafe	8	15	1	3	1	8	8	9
Highest Dose Deemed too Safe	0	0	0	0	0	0	0	0
Precision	0	16	10	40	96	0	0	0
Hard Safety	1	0	1	2	1	0	0	0
Maximum Patients	3	2	1	1	0	6	1	1
Unsafe (Total)	8	15	1	4	2	8	8	9
Sufficient/Precision (Total)	91	85	99	95	98	90	92	90
Stopping Reason				*Scenario 16*				
Sufficient Information	15	4	34	11	2	20	20	19
Lowest Dose Deemed Unsafe	85	96	66	86	95	80	80	81
Highest Dose Deemed too Safe	0	0	0	0	0	0	0	0
Precision	0	10	0	0	10	0	0	0
Hard Safety	60	23	66	47	47	24	24	24
Maximum Patients	0	0	0	0	0	0	0	0
Unsafe (Total)	85	96	66	89	96	80	80	81
Sufficient/Precision (Total)	15	4	34	11	4	20	20	19
Stopping Reason				*Scenario 17 (D)*				
Sufficient Information	96	64	63	5	8	99	99	99
Lowest Dose Deemed Unsafe	0	1	0	0	0	1	1	1
Highest Dose Deemed too Safe	0	0	0	0	0	0	0	0
Precision	0	44	60	99	100	0	0	0
Hard Safety	0	0	0	0	0	0	0	0
Maximum Patients	13	4	0	0	0	1	1	1
Unsafe (Total)	0	1	0	0	0	1	1	1
Sufficient/Precision (Total)	96	98	100	100	100	99	99	99

Table 16: Setting 2: Summary of stopping reasons for Setting 2 (Scenarios 13-17), expressed as a percentage of simulations where the given stopping rule was triggered. The sum of these may be greater than 100, since it is possible for more than one rule to be triggered in a single trial.

4 Model-Assisted Designs using Original Hyper-Parameters

	TITEBOIN	TITE_mTPI2	R_mTPI2
1 (A)	48	45	48
2	38	32	36
3	34	29	30
4	29	26	23
5	16	16	8
6	7	14	5
7(B)	17	21	10
8	39	28	36
9 (C)	44	52	53
10	38	31	37
11	36	35	39
12	15	18	8
13	72	72	64
14	27	28	19
15	53	47	49
16	74	80	81
17 (D)	96	97	96
Mean	40	39	38

Table 17: Setting 2: Percentage of simulations correctly identifying MTD for model-assisted designs with original parameters.

	TITEBOIN	TITE_mTPI2	R_mTPI2
Scenario	*Mean Duration in Weeks (sd)*		
1 (A)	23 (12)	42 (27)	39 (29)
2	30 (14)	59 (30)	60 (32)
3	39 (13)	78 (27)	84 (29)
4	47 (14)	95 (24)	104 (25)
5	51 (15)	103 (24)	114 (27)
6	52 (13)	110 (19)	122 (21)
7 (B)	39 (17)	79 (33)	84 (38)
8	42 (12)	85 (25)	92 (25)
9 (C)	18 (11)	32 (27)	26 (24)
10	43 (13)	87 (25)	95 (26)
11	36 (14)	72 (30)	76 (33)
12	51 (15)	104 (24)	115 (27)
13	46 (12)	103 (19)	115 (23)
14	44 (15)	89 (28)	98 (32)
15	31 (11)	58 (22)	63 (27)
16	12 (7)	16 (13)	13 (12)
17 (D)	41 (7)	76 (10)	92 (13)
Mean	38	76	82
Scenario	*Mean Number of Patients (sd)*		
1 (A)	11 (6)	13 (7)	11 (6)
2	15 (6)	17 (7)	16 (7)
3	19 (6)	21 (5)	21 (6)
4	22 (5)	24 (4)	24 (5)
5	24 (5)	24 (5)	26 (5)
6	25 (4)	24 (4)	26 (4)
7 (B)	19 (7)	21 (7)	20 (8)
8	21 (5)	22 (4)	22 (5)
9 (C)	9 (5)	10 (7)	8 (6)
10	21 (5)	23 (4)	23 (5)
11	18 (7)	20 (6)	19 (7)
12	24 (5)	24 (5)	26 (5)
13	22 (5)	21 (5)	24 (5)
14	21 (6)	23 (5)	23 (6)
15	16 (5)	17 (6)	16 (6)
16	6 (3)	6 (4)	5 (3)
17 (D)	20 (3)	20 (3)	21 (3)
Mean	18	19	19

Table 18: Setting 2: Measures of size of the trial across scenarios, mean and sd of duration and number of patients for model-assisted designs with original parameters.

	**TITEBOIN**	**TITE_mTPI2**	**R_mTPI2**
Scenario	*Mean Number of Patients on MTD (sd)*		
1 (A)	7 (3)	6 (3)	7 (3)
2	5 (4)	6 (4)	6 (4)
3	5 (4)	6 (4)	6 (4)
4	5 (4)	5 (3)	5 (4)
5	4 (4)	5 (4)	4 (4)
6	3 (3)	4 (4)	3 (3)
7 (B)	3 (4)	4 (4)	3 (4)
8	6 (3)	6 (3)	7 (3)
9 (C)	NA (NA)	NA (NA)	NA (NA)
10	6 (3)	6 (3)	7 (3)
11	6 (3)	6 (3)	6 (3)
12	4 (4)	5 (4)	4 (4)
13	NA (NA)	NA (NA)	NA (NA)
14	5 (4)	5 (4)	5 (4)
15	7 (3)	7 (3)	7 (3)
16	NA (NA)	NA (NA)	NA (NA)
17 (D)	9 (1)	9 (1)	9 (1)
Mean	5	6	6
Scenario	*Mean Number of Patients on Unsafe Doses (sd)*		
1	5 (6)	7 (7)	4 (6)
2	4 (5)	6 (7)	3 (5)
3	3 (5)	5 (6)	3 (5)
4	3 (4)	5 (5)	3 (4)
5	2 (3)	3 (3)	1 (3)
6	0 (0)	0 (0)	0 (0)
7	2 (3)	3 (5)	1 (3)
8	5 (6)	7 (7)	4 (6)
9	9 (5)	10 (7)	8 (6)
10	6 (6)	8 (7)	5 (6)
11	7 (7)	9 (7)	7 (6)
12	2 (3)	3 (3)	2 (3)
13	0 (0)	0 (0)	0 (0)
14	2 (4)	4 (5)	2 (3)
15	3 (4)	5 (5)	3 (4)
16	6 (3)	6 (4)	5 (3)
17	5 (2)	5 (2)	5 (2)
Mean	4	5	3

Table 19: Setting 2: Mean and standard deviation of the number of patients assigned to the true MTD and to unsafe doses for model-assisted designs with original parameters.

	TITEBOIN	TITE_mTPI2	R_mTPI2
Stopping Reason		*Scenario 1 (A)*	
Sufficient Information	74	68	67
Lowest Dose Deemed Unsafe	26	31	32
Highest Dose Deemed too Safe	0	0	0
Precision	0	0	0
Hard Safety	6	4	4
Maximum Patients	0	2	0
Unsafe (Total)	26	31	32
Sufficient/Precision (Total)	74	68	67
Stopping Reason		*Scenario 2*	
Sufficient Information	88	84	83
Lowest Dose Deemed Unsafe	11	13	15
Highest Dose Deemed too Safe	0	0	0
Precision	0	0	0
Hard Safety	1	1	1
Maximum Patients	2	6	3
Unsafe (Total)	11	13	15
Sufficient/Precision (Total)	88	84	83
Stopping Reason		*Scenario 3*	
Sufficient Information	94	91	88
Lowest Dose Deemed Unsafe	3	3	3
Highest Dose Deemed too Safe	0	0	0
Precision	0	0	0
Hard Safety	0	0	0
Maximum Patients	7	10	12
Unsafe (Total)	3	3	3
Sufficient/Precision (Total)	94	91	88
Stopping Reason		*Scenario 4*	
Sufficient Information	90	87	79
Lowest Dose Deemed Unsafe	1	1	1
Highest Dose Deemed too Safe	2	4	1
Precision	0	0	0
Hard Safety	0	0	0
Maximum Patients	17	16	26
Unsafe (Total)	1	1	1
Sufficient/Precision (Total)	90	87	79
Stopping Reason		*Scenario 5*	
Sufficient Information	77	80	63
Lowest Dose Deemed Unsafe	1	1	1
Highest Dose Deemed too Safe	9	12	6
Precision	0	0	0
Hard Safety	0	0	0
Maximum Patients	25	21	40
Unsafe (Total)	1	1	1
Sufficient/Precision (Total)	77	80	63
Stopping Reason		*Scenario 6*	
Sufficient Information	59	66	47
Lowest Dose Deemed Unsafe	0	0	0
Highest Dose Deemed too Safe	30	29	23
Precision	0	0	0
Hard Safety	0	0	0
Maximum Patients	26	23	41
Unsafe (Total)	0	0	0
Sufficient/Precision (Total)	59	66	47

Table 20: Setting 2: Summary of stopping reasons for Setting 2 (Scenarios 1-6), expressed as a percentage of simulations where the given stopping rule was triggered for model-assisted designs with original parameters. The sum of these may be greater than 100, since it is possible for more than one rule to be triggered in a single trial.

	TITEBOIN	TITE_mTPI2	R_mTPI2
Stopping Reason		*Scenario 7 (B)*	
Sufficient Information	88	86	77
Lowest Dose Deemed Unsafe	6	6	8
Highest Dose Deemed too Safe	0	1	0
Precision	0	0	0
Hard Safety	1	0	0
Maximum Patients	11	12	20
Unsafe (Total)	6	6	8
Sufficient/Precision (Total)	88	86	77
Stopping Reason		*Scenario 8*	
Sufficient Information	94	90	88
Lowest Dose Deemed Unsafe	1	1	1
Highest Dose Deemed too Safe	1	3	1
Precision	0	0	0
Hard Safety	0	0	0
Maximum Patients	9	12	14
Unsafe (Total)	1	1	1
Sufficient/Precision (Total)	94	90	88
Stopping Reason		*Scenario 9 (C)*	
Sufficient Information	55	47	47
Lowest Dose Deemed Unsafe	44	52	53
Highest Dose Deemed too Safe	0	0	0
Precision	0	0	0
Hard Safety	15	10	11
Maximum Patients	0	2	0
Unsafe (Total)	44	52	53
Sufficient/Precision (Total)	55	47	47
Stopping Reason		*Scenario 10*	
Sufficient Information	94	90	87
Lowest Dose Deemed Unsafe	1	1	1
Highest Dose Deemed too Safe	1	2	0
Precision	0	0	0
Hard Safety	0	0	0
Maximum Patients	10	13	17
Unsafe (Total)	1	1	1
Sufficient/Precision (Total)	94	90	87
Stopping Reason		*Scenario 11*	
Sufficient Information	91	88	85
Lowest Dose Deemed Unsafe	6	6	8
Highest Dose Deemed too Safe	0	1	0
Precision	0	0	0
Hard Safety	1	0	0
Maximum Patients	6	9	9
Unsafe (Total)	6	6	8
Sufficient/Precision (Total)	91	88	85
Stopping Reason		*Scenario 12*	
Sufficient Information	74	79	59
Lowest Dose Deemed Unsafe	1	1	1
Highest Dose Deemed too Safe	11	13	8
Precision	0	0	0
Hard Safety	0	0	0
Maximum Patients	27	22	42
Unsafe (Total)	1	1	1
Sufficient/Precision (Total)	74	79	59

Table 21: Setting 2: Summary of stopping reasons for Setting 2 (Scenarios 7-12), expressed as a percentage of simulations where the given stopping rule was triggered for model-assisted designs with original parameters. The sum of these may be greater than 100, since it is possible for more than one rule to be triggered in a single trial.

	TITEBOIN	TITE_mTPI2	R_mTPI2
Stopping Reason		*Scenario 13*	
Sufficient Information	23	25	19
Lowest Dose Deemed Unsafe	1	1	1
Highest Dose Deemed too Safe	72	72	64
Precision	0	0	0
Hard Safety	0	0	0
Maximum Patients	11	11	25
Unsafe (Total)	1	1	1
Sufficient/Precision (Total)	23	25	19
Stopping Reason		*Scenario 14*	
Sufficient Information	90	88	78
Lowest Dose Deemed Unsafe	3	3	3
Highest Dose Deemed too Safe	1	1	0
Precision	0	0	0
Hard Safety	0	0	0
Maximum Patients	15	14	25
Unsafe (Total)	3	3	3
Sufficient/Precision (Total)	90	88	78
Stopping Reason		*Scenario 15*	
Sufficient Information	93	91	90
Lowest Dose Deemed Unsafe	7	8	9
Highest Dose Deemed too Safe	0	0	0
Precision	0	0	0
Hard Safety	1	0	0
Maximum Patients	1	2	1
Unsafe (Total)	7	8	9
Sufficient/Precision (Total)	93	91	90
Stopping Reason		*Scenario 16*	
Sufficient Information	26	20	19
Lowest Dose Deemed Unsafe	74	80	81
Highest Dose Deemed too Safe	0	0	0
Precision	0	0	0
Hard Safety	33	24	24
Maximum Patients	0	0	0
Unsafe (Total)	74	80	81
Sufficient/Precision (Total)	26	20	19
Stopping Reason		*Scenario 17 (D)*	
Sufficient Information	99	99	99
Lowest Dose Deemed Unsafe	1	1	1
Highest Dose Deemed too Safe	0	0	0
Precision	0	0	0
Hard Safety	0	0	0
Maximum Patients	1	1	2
Unsafe (Total)	1	1	1
Sufficient/Precision (Total)	99	99	99

Table 22: Setting 2: Summary of stopping reasons for Setting 2 (Scenarios 13-17), expressed as a percentage of simulations where the given stopping rule was triggered for model-assisted designs with original parameters. The sum of these may be greater than 100, since it is possible for more than one rule to be triggered in a single trial.
